# Gastroenteritis at a University in Texas: An Epidemiologic Case Study

**DOI:** 10.3201/eid1207.060457

**Published:** 2006-07

**Authors:** Philip S. Brachman

**Affiliations:** * Rollins School of Public Health, Atlanta, Georgia, USA

**Keywords:** Gastroenteritis, book review, CDC, field case, training aid

This CD-ROM is an important addition to case exercises in field epidemiology that serve to educate when actual participation in a field investigation is not possible or practical. The authors have prepared a case exercise based on an actual field investigation with real data that have been put together in a meaningful and effective way. The use of an epidemic of gastroenteritis is a cutting-edge element, since foodborne disease is a major public health problem today. The outbreak occurs on a college campus, which lends an air of verisimilitude, and the causative agent, norovirus, is a genuine public health threat.

This reviewer had a number of specific editorial recommendations for the authors that could enhance forthcoming versions. These suggestions included inserting a case definition in the investigation outline; adding the role of the state laboratory; consistently labeling outbreak, epidemic, and epidemic curve throughout the program; clarifying the rationale for limiting the outbreak to the university; further refining methods for the study and controls; using 2×2 tables to illustrate epidemiologic ratios; and expanding the employee training plan.

Overall, these types of training aids are needed as we attempt to further expose public health workers to field investigations so that they can conduct investigations effectively. The reference to additional educational material throughout the steps is a well-conceived and appropriate aspect of the investigation. The narrative information, questions, and explanations are appropriate and flow smoothly.

